# Correction-free force calibration for magnetic tweezers experiments

**DOI:** 10.1038/s41598-018-34360-4

**Published:** 2018-10-29

**Authors:** Eugen Ostrofet, Flávia Stal Papini, David Dulin

**Affiliations:** Junior Research Group 2, Interdisciplinary Center for Clinical Research, Friedrich Alexander University Erlangen-Nürnberg (FAU), Hartmannstr. 14, 91052 Erlangen, Germany

## Abstract

Magnetic tweezers are a powerful technique to perform high-throughput and high-resolution force spectroscopy experiments at the single-molecule level. The camera-based detection of magnetic tweezers enables the observation of hundreds of magnetic beads in parallel, and therefore the characterization of the mechanochemical behavior of hundreds of nucleic acids and enzymes. However, magnetic tweezers experiments require an accurate force calibration to extract quantitative data, which is limited to low forces if the deleterious effect of the finite camera open shutter time (*τ*_*sh*_) is not corrected. Here, we provide a simple method to perform correction-free force calibration for high-throughput magnetic tweezers at low image acquisition frequency (*f*_*ac*_). By significantly reducing *τ*_*sh*_ to at most 1/4 the characteristic time of the tethered magnetic bead, we accurately evaluated the variance of the magnetic bead position along the axis parallel to the magnetic field, estimating the force with a relative error of ~10% (standard deviation), being only limited by the bead-to-bead difference. We calibrated several magnets - magnetic beads configurations, covering a force range from ~50 fN to ~60 pN. In addition, for the presented configurations, we provide a table with the mathematical expressions that describe the force as a function of the magnets position.

## Introduction

In the recent years, single-molecule force spectroscopy techniques, e.g. atomic force microscopy (AFM), optical tweezers and magnetic tweezers^1,2^, have been successfully used to provide insights on molecular complexes, allowing the direct observation of the catalytic activity of enzymes or the conformation of molecules, e.g. nucleic acids and proteins, at the single-molecule level. Magnetic tweezers are a force and torque spectroscopy technique^3–6^ that operate naturally as a force clamp and apply a homogenous force from ~10 fN to ~1 nN over a large field of view^1,7^. Magnetic tweezers have been used in different settings to study nucleic acids - protein interactions, e.g. topoisomerase^8–10^, gyrase^11,12^, phage replication^13–15^, bacterial^16–18^ and viral transcription^19–21^, helicase unwinding activity^22–24^, CRISPR-Cas interaction^25^, chromatin assembly^26,27^, and cell mechanics^28–30^.

The success of the magnetic tweezers assay comes from its simplicity: it is basically a magnetic element – either permanent magnet(s) or an electromagnet – placed above a flow chamber^31^. An infinitely corrected microscope objective is mounted on an inverted microscope and images the sample within the flow chamber onto a CCD or a CMOS camera (Fig. 1a). Each nucleic acid is attached at one end to the bottom surface of the flow chamber and at the other end to a force transducer, i.e. a micron size superparamagnetic bead (or simply magnetic bead) (Fig. 1a, inset)^32,33^. Because of the CMOS camera-based detection, magnetic tweezers offer simultaneously high parallelization^2,34–36^ and high spatiotemporal resolution^37,38^ capabilities.

**Figure 1:**
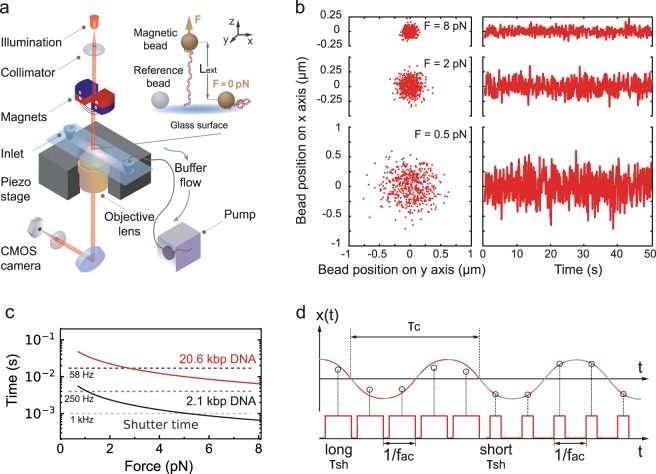
Magnetic tweezers assay and experimental characteristics. (**a**) A magnetic tweezers setup consists of an inverted microscope, a flow chamber and a pair of permanent magnets (represented here vertically aligned, as used in the experiments). In the flow chamber, the nucleic acid molecules are attached at one end to a magnetic bead and at the other end to the flow chamber bottom glass coverslip top surface. The magnets are placed above the flow cell and decreasing their distance to the magnetic beads increases the force applied to the tether. A collimated light source located above the magnets illuminates the flow chamber through the magnets gap. The light is collected by an oil immersion objective underneath the flow chamber and the field of view is imaged onto a complementary metal-oxide-semiconductor (CMOS) camera (Material and Methods). (**b**) Lateral displacements of a MyOne bead tethered to the 20.6 kbp DNA construct at three different forces (8 pN, 2 pN and 0.5 pN). (**c**) The characteristic timescale (*t*_*c*,*x*_) of the fluctuations along the x-axis of a MyOne magnetic bead tethered by either a 20.6 kbp (red) or a 2.1 kbp (black) DNA construct as a function of the applied force (Material and Methods, Equation (2)). The dashed grey lines indicate the shutter time (*τ*_*sh*_) of the camera for different acquisition frequencies (58 Hz, 250 Hz and 1 kHz). (**d**) Illustration of the effect of long and short *τ*_*sh*_ on the estimation of the position of a system with a defined characteristic time *τ*_*c*_ at constant *f*_*ac*_.

The nucleic acid tethered magnetic bead system is well described by an inverted pendulum model, where the nucleic acid experiences a force *F*^37,39,40^ and the magnetic bead position fluctuates around its average position from the collision of the water molecules of the surrounding buffer, i.e. a Brownian motion. In these settings, the tethered magnetic bead is described by the variance of its position along the axis parallel to the magnetic field *δx*^2^, linked to *F* (Fig. 1b) by the equipartition theorem^6^:

1 $$\langle \delta {x}^{2}\rangle =\frac{{k}_{B}T{L}_{ext}}{F},$$

where *k*_*B*_ is the Boltzmann constant, *T* is the absolute temperature of the surrounding buffer and *L*_*ext*_ is the extension of the tether at the force *F*, and by its characteristic time along the same axis *t*_*c*,*x*_ (Fig. 1c):

2 $${t}_{c,x}=\frac{\gamma }{{k}_{x}}=\frac{6\pi \eta R{L}_{ext}}{F},$$

where *γ* is the drag coefficient of the bead, *k*_*x*_ the stiffness of the trap along the x-axis, *η* is the viscosity of the medium and *R* the radius of the bead. Force calibration is an essential pre-requisite to any biological experiment that uses magnetic tweezers. Equation (1) is usually not the most favorable method to measure the force in magnetic tweezers experiments^39,41–44^ because the finite camera open shutter time (*τ*_*sh*_) averages the Brownian fluctuations when *τ*_*sh*_ is comparable to *t*_*c*,*x*_. This effect, called camera image blurring, artificially reduces *δx*^2^, which leads to force overestimation^45,46^. In other words, force calibration using Equation (1) is limited to long *t*_*c*,*x*_, i.e. long tethers and low force^6^ (Material and Methods, Equation (2)). Though magnetic tweezers that acquire images at high *f*_*ac*_ have now been demonstrated^37,38,47^, this is at the cost of the instrument high parallelization. Therefore, to correct the camera image blurring effect in magnetic tweezers force estimation at low *f*_*ac*_, several studies have been published, where bias-corrections were introduced using either the power spectral density analysis^3,39,41–43^, or the Allan variance of the bead position^41^. These analyses are performed with data acquired in zero dead time condition, i.e. ${\tau }_{sh}\sim 1/{f}_{ac}$. Though powerful, frequency domain and time domain image blurring corrected methods are relatively complex to implement computationally^41,42^, and are not available in non-proprietary programming language, e.g. Python.

In this study, we describe the experimental conditions to achieve a direct and correction-free force calibration for magnetic tweezers experiments using Equation (1) and non-zero dead time data acquisition, i.e. ${\tau }_{sh} < 1/{f}_{ac}$. Though this method has been punctually applied in different settings^42,48^, the experimental conditions to achieve accurate force calibration at ${\tau }_{sh} < 1/{f}_{ac}$ have not yet been described. Taking advantage of a low-cost and bright LED source, we show here that by having *τ*_*sh*_ ~ 4-fold smaller than *t*_*c*,*x*_, while keeping the illumination intensity on the camera constant, Equation (1) is directly applicable to extract the force with an accuracy only limited by the bead-to-bead difference in magnetic content, i.e. 10% standard deviation (s.d.) in force. We apply our calibration strategy for a wide range of forces, i.e. from ~50 fN to ~60 pN, with two types of superparamagnetic beads, two different magnets configurations, and a long and a short DNA construct. We further demonstrate the consistency of our method by comparing our results with a previously published force calibration study^42^, which shows consistency in the evaluated force ranges. Furthermore, our data agree with theoretical prediction, numerical simulations and with the magnetic content difference between the two magnetic beads we have studied here. Our study offers a simple and robust method to calibrate forces in magnetic tweezers experiments, particularly when using a very large field of view CMOS camera with a low acquisition frequency, and is easily applicable to other camera-based high-throughput force spectroscopy techniques, such as stretch-flow assays^49^ and acoustic-force spectroscopy^50^.

## Results

### Spatiotemporal resolution and drift correction

In the magnetic tweezers force calibration study we present here, the magnetic field is generated by a pair of permanent magnets vertically aligned with a gap of either 1 mm or 0.3 mm (Fig. 1a, Material and Methods). The DNA construct is attached at one end to the coverslip surface and at the other end to the magnetic bead (Material and Methods). The large field of view provided by the CMOS camera, i.e. ~0.5 × 0.4 mm, offers the possibility to observe hundreds of magnetic beads, i.e. nucleic acids molecules, at the single-molecule level^2,21,36^. To correct for the mechanical drift of the flow chamber, we subtract the position of the reference bead attached to the surface of the coverslip to the tethered magnetic beads positions (Fig. 1a). Since the force calibration we perform here relies on the equipartition theorem (Equation (1)), we need to evaluate whether the resolution of our assay along the x-axis – for *δx*^2^ estimation – and the z-axis – for *L*_*ext*_ estimation – is sufficient for such force calibration. It has been previously shown that the bead size at a constant pixel size and the illumination intensity strongly affect the tracking resolution of a magnetic tweezers assay^37,51^. We therefore evaluated the tracking noise versus the thermal noise using the Allan deviation (AD)^37,41^ for the two reference beads and tethered magnetic beads, while keeping the illumination intensity at ~160 grey levels (256 total) (Supplementary Fig. 1). The AD is defined as one-half the average difference in bead position between adjacent intervals of duration *τ*_*AD*_ over all intervals of length *τ*_*AD*_^52^. In short, the AD provides an estimation of the spatiotemporal resolution of the assay in the time domain. We use here the fully overlapping sampling strategy to evaluate the AD^41^. For *τ*_*AD*_ = 0.1 s, i.e. the standard deviation at 1 frame, we measure along the x-axis an AD of 1.5 nm and 4 nm for 3 µm and 1.1 µm diameter reference beads, respectively (Supplementary Fig. 1a,c). The tracking noise is similarly low along the z-axis (Supplementary Fig. 1b,d). Comparing the tether fluctuations along the x-axis at the highest attainable force using 1 mm gap vertically aligned magnets at *τ*_*AD*_ = 0.1 s for the M270 (Supplementary Fig. 1a) and the MyOne magnetic beads (Supplementary Fig. 1c) to the tracking noise, we measure that the thermal noise is at least 20-fold larger than the tracking noise. Similar observations have been made for the z-axis when using M270 (Supplementary Fig. 1b) and MyOne (Supplementary Fig. 1d) magnetic beads. Small open camera shutter time duration leads to an increase in tracking noise if the illumination intensity is not kept constant. Indeed, the tracking resolution decreases as one over the square root of the illumination intensity^37^. Therefore, we can extrapolate that the tracking noise at, e.g. 20 grey levels –a very low illumination intensity–, from Supplementary Fig. 1c, and we estimate the variance of the tracking noise to be ~128 nm^2^ for a 1.1 µm diameter reference bead. Given that the thermal noise variance along the x-axis of a magnetic bead tethered by a 20.6 kbp long DNA molecule experiencing a 8 pN force is ~3600 nm^2^, the force is underestimated by ~3.5%. Similarly for the M270 magnetic beads (Supplementary Fig. 1a), we extrapolate the variance of the tracking noise along the x-axis at 20 grey levels to be ~18 nm^2^, while the tethered magnetic bead position variance along the x-axis and at ~60 pN is ~480 nm^2^, leading to a force underestimation of ~3.8%. Overall, neither the mechanical drift nor the tracking resolution is limiting to perform a force estimation using Equation (1) in our assay.

### Effect of the camera open shutter time on the force estimation

The variance of the bead position decreases when the force increases (Equation (1)), which is illustrated in Fig. 1b. Furthermore, *t*_*c*,*x*_ also decreases with the increase in force and decrease in tether length (Material and Methods, Equation (2); Fig. 1c). Interestingly,*t*_*c*,_ is in the range of *τ*_*sh*_ for standard cameras, i.e. *f*_*ac*_ ~ 50–100 Hz and ${\tau }_{sh}\sim 1/{f}_{ac}$ (Fig. 1c). Consequently, camera-based detection leads to image blurring, and averages out the beads trajectory (Fig. 1d). Therefore, a value of *τ*_*sh*_ close to *t*_*c*,*x*_ results in underestimating the variance of the bead position and in overestimating the force^53^.

To quantify the effect of *τ*_*sh*_ in the force calibration, we vary *τ*_*sh*_ from 0.5 to 20 ms and estimate the force using Equation (1) at different magnets position, 0 mm being the top surface of the upper coverslip of the flow chamber (Fig. 2a,b). We have only selected those points in the data set that are separated by at least more than one *t*_*c*,*x*_, to keep the subsequent bead position estimates uncorrelated. By doing so, we have considerably reduced the size of the data set, while maintaining the reduction in the force statistical error following $1/\sqrt{N}$, *N* being the dimension of the data set. Using the 20.6 kbp dsDNA construct, we observe a similar trend with M270 (Fig. 2a) and MyOne (Fig. 2b) magnetic beads: the force estimations overlap at small *τ*_*sh*_, i.e. 0.5 and 1 ms, and significantly diverge with increasing *τ*_*sh*_ for magnets positions below ~2.5 mm. For both types of beads, the discrepancy from the best force estimation grows as a function of the applied force, which agrees well with the decrease of *t*_*c*,*x*_ (Equation (2)). For the 1 mm gap vertical orientation magnets configuration, 0.1 mm magnets distance from the top coverslip and M270 magnetic beads, we estimate the force to be (60 ± 4) pN (mean ± standard deviation) at *τ*_*sh*_ = 0.5 ms, and (179 ± 7) pN for *τ*_*sh*_ = 20 ms, i.e. a ~3-fold difference (Fig. 2a). For MyOne beads, in similar magnets configuration, we estimate forces of (8.6 ± 1.1) pN at *τ*_*sh*_ = 0.5 ms and (14.1 ± 2.1) pN at *τ*_*sh*_ = 20 ms, i.e. ~1.7-fold difference (Fig. 2b). Investigating how the force estimation varies within a bead population at different *τ*_*sh*_, we observe that the error in the force estimation is independent of *τ*_*sh*_ (Supplementary Fig. 2a,b) and lies within the bead-to-bead variation of the force estimation, as previously reported for MyOne and M270 magnetic beads^7,33^, i.e. ~10% (s.d.) (Supplementary Fig. 2).

**Figure 2:**
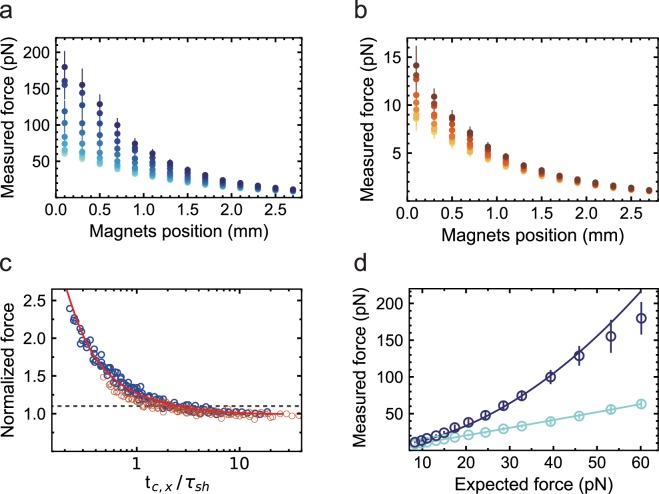
Experimental readout and simulation of the effect of the shutter time on the measured force. Force measurement as a function of the distance of the vertically aligned pair of permanent magnets from the flow cell top surface with (**a**) M270 (2.8 µm diameter) and (**b**) MyOne (1 µm diameter) magnetic beads tethered by the 20.6 kbp dsDNA construct and with *τ*_*sh*_ equal to 0.5, 1, 2, 3, 5, 8, 11, 14, 17 and 20 ms (color code from light to dark). n = 12–16 for M270 magnetic beads and n = 15–21 for MyOne magnetic beads, depending on the *τ*_*sh*_ experiment. (**c**) Normalized force at a given *τ*_*sh*_ by the force at *τ*_*sh*_ = 0.5 ms for the same magnets pair position as a function of ${t}_{c,x}/{\tau }_{sh}\,$ for M270 and MyOne magnetic beads (blue and orange circles, respectively). The dashed line represents the 10% limit in force overestimation. The solid red line is a plot from Equation (3) (Materials and Methods). The solid red line is (**d**) Measured force from experiments (circles) and numerical simulation (solid lines, Material and Methods) as a function of the expected force, for the 20.6 kbp DNA construct, M270 magnetic beads, a 1 mm gap vertically aligned magnets configuration, and either *τ*_*sh*_ = 1 ms (light blue) or *τ*_*sh*_ = 20 ms (dark blue). The data were acquired at *f*_*ac*_ = 10 Hz. The error bars in the plot are one standard deviation.

To illustrate the relationship between *τ*_*sh*_ and *t*_*c*,*x*_ with the force estimation using Equation (1), the force estimated at a given *τ*_*sh*_ is normalized by the force estimated at *τ*_*sh*_ = 0.5 ms, i.e. $F({\tau }_{sh})/F({\tau }_{sh}=0.5\,ms)$, for each magnet position, and is represented as a function of ${t}_{c,x}/{\tau }_{sh}$ (Fig. 2c). In other words, Fig. 2c shows the force overestimation as a function of ${t}_{c,x}/{\tau }_{sh}$. For example, for ${t}_{c,x}/{\tau }_{sh}\sim 4$, we measure a ~10% overestimation for both M270 and MyOne magnetic beads (dashed line in Fig. 2c). The discrepancy between the measured force and the expected force as a function of ${t}_{c,x}/{\tau }_{sh}\,$ is well described analytically by the bias introduced on the bead position estimation by the camera image blurring (Fig. 2c, Equation (3) in Materials and Methods).

To determine whether the theory confirms our experimental observations, we have simulated the position of a 20.6 kbp DNA-tethered M270 magnetic bead under force and we add the camera image blurring effect at *τ*_*sh*_ = 1 and *τ*_*sh*_ = 20 ms^37,54^ in the simulation (Material and Methods). The model does not take into account the deleterious effects of the tether side attachment on the magnetic bead^43,55^. For both M270 and MyOne magnetic beads, we find a good agreement between the force estimation obtained from the numerical simulation and from the experiments for both *τ*_*sh*_ (Fig. 2d, Supplementary Fig. 3).

### Calibration and force extension

Using the 20.6 kbp dsDNA, MyOne magnetic beads, 1 mm gap vertically oriented magnets configuration, and *τ*_*sh*_ = 0.5 ms, we have estimated the force for magnets positions varying from 0.1 to 7.5 mm (Fig. 3a) (Material and Methods). As expected, the force decays bi-exponentially with the magnets position^33,42^ (Supplementary Fig. 4). For forces above ~0.3 pN, our calibration is in excellent agreement with a previously published calibration method based on spectral correction^42^, whereas a discrepancy is observed for force estimation points below ~0.3 pN (Fig. 3a), which arises from a diverging bi-exponential fitting procedure at low force. Comparison of our force estimation using the M270 magnetic beads and 1 mm gap vertically oriented magnets configuration shows a similar trend as with previous estimations^42^ (Fig. 3a). Furthermore, the difference in force observed for MyOne and M270 magnetic beads at the same magnets position agrees well with the difference in magnetic content of these beads (specifications from Invitrogen, Thermofisher, Germany), i.e. M270 magnetic beads contain ~12-times more magnetic material than MyOne magnetic beads, which translates into a ~8-fold force difference in favor of M270 magnetic beads (Fig. 3a). We have also estimated the force for the 0.3 mm gap vertically oriented magnets configuration and MyOne magnetic beads for either thin or thick flow chambers (Material and Methods), and both data sets overlap well for equivalent magnets distances to the beads (Fig. 3b). The bi-exponential expressions of the force versus magnets distance for the different magnets and magnetic beads configurations we have evaluated here are listed in Table 1.

**Figure 3:**
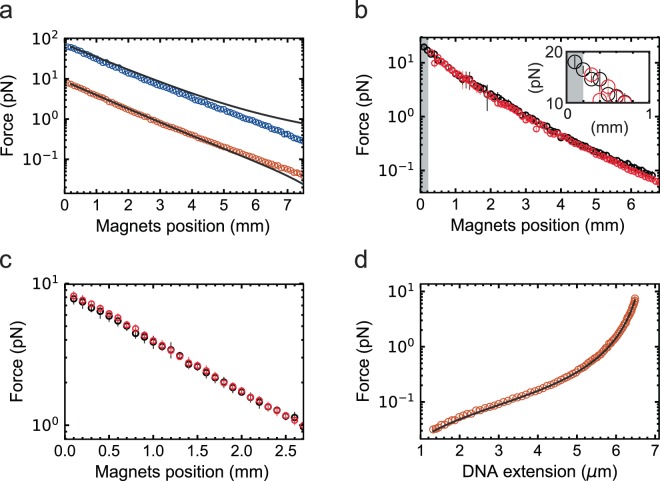
Force calibration at 0.5 ms shutter time. (**a**) Forces obtained directly from the variance of the bead fluctuation acquired at *τ*_*sh*_ = 0.5 ms (circles) plotted against the magnets position for MyOne (orange) and M270 (blue) magnetic beads using a 1 mm gap vertically aligned magnets configuration. The solid line corresponds to the bi-exponential expression from Yu and co-workers^42^ from a force calibration performed in the same experimental configuration. (**b**) Force measurements obtained directly from the variance of the bead fluctuation acquired at *τ*_*sh*_ = 0.5 ms using a 0.3 mm gap vertically aligned magnets configuration and MyOne magnetic beads with either a thin (black circles) or a thick (red circles) flow chamber. The shaded area is the extra accessible region when performing the measurements with a thin flow cell. The gain in accessible force from using a thin flow cell is highlighted in the inset. (**c**) Forces obtained directly from the equipartition theorem, with either *τ*_*sh*_ = 0.2 ms and a 2.1 kbp DNA construct (black circles), or *τ*_*sh*_ = 0.5 ms and a 20.6 kbp DNA construct (red circles), both using a 1 mm gap vertically aligned magnets configuration, MyOne magnetic beads and 3 µm reference beads (Material and Methods). (**d**) Force represented as a function of the 20.6 kbp DNA construct extension (red circles); n = 11. The solid line is a fit with the inextensible worm-like chain model^56^, i.e. $F({L}_{ext})=\frac{{k}_{B}T}{{L}_{p}}(\frac{1}{4{(1-{L}_{ext}/{L}_{c})}^{2}}-\frac{1}{4}+\frac{{L}_{ext}}{{L}_{c}})$, with the contour length *L*_*c*_ and the persistence length *L*_*p*_ as free parameters. From the fit, we extracted *L*_*c*_ = (6.9 ± 0.1) µm and *L*_*p*_ = (44.0 ± 0.1) nm. The data were acquired at *f*_*ac*_ = 10 Hz. The error bars in the plot represent one standard deviation.

**Table 1:** Bi-exponential expression of the force F (pN) as a function of the magnets distance (in mm) from the top cover slip x for several experimental configurations.

Index	Fit to a double exponential	Bead	Magnets gap	Flow cell thickness
1	$F=22.3811\ast {e}^{-1.4578\ast x}+52.2987\ast {e}^{-0.6912\ast x}$	M270	1 mm	0.4 mm
2	$F=5.7061\ast {e}^{-1.0203\ast x}+3.1215\ast {e}^{-0.5843\ast x}$	MyOne	1 mm	0.4 mm
3	$F=4.6686\ast {e}^{-0.6645\ast x}+11.9429\ast {e}^{-1.4137\ast x}$	MyOne	0.3 mm	0.4 mm
4	$F=17.5310\ast {e}^{-1.1649\ast x}+2.7214\ast {e}^{-0.5527\ast x}$	MyOne	0.3 mm	0.2 mm

The flow cell thickness excludes the bottom coverslip thickness. The numerical expression covers magnets distances from 0.1 to 7.5 mm, and are extracted from Supplementary Fig. 4.

Since *t*_*c*,*x*_ decreases linearly with *L*_*ext*_ (Material and Methods, Equation (2)), we have evaluated the effect of using a short tether on the force estimation, expecting that such calibration will become particularly difficult without camera image blurring correction at high forces. For example, for MyOne magnetic beads and ~8 pN applied force, *t*_*c*,*x*_ varies from ~8 ms to ~0.8 ms when using the 20.6 kbp and 2.1 kbp DNA constructs, respectively. Using these two DNA constructs, we have compared the force estimation using MyOne magnetic beads, 1 mm gap vertically aligned magnets configuration, and *τ*_*sh*_ = 0.2 ms (Fig. 3c). We observe that both measurements agree within the error bars (one standard deviation, Fig. 3c), validating the equipartition theorem (Equation (1)) at small *τ*_*sh*_ force calibration approach, even for small *t*_*c*,*x*_.

To further evaluate the accuracy of our force calibration, we represent the extension of the 20.6 kbp dsDNA construct as a function of the applied force (Fig. 3d). The resulting force-extension curve is accurately described by the inextensible Worm-Like Chain (WLC) model^56^, providing a contour length of (6.9 ± 0.1) µm and a persistence length of (44 ± 0.1) nm, which are in agreement with the literature for such a DNA construct^4^.

## Discussion

In this study, we provide the experimental conditions to achieve a correction-free force calibration with magnetic tweezers applying the equipartition theorem (Equation (1)). The attractiveness of this approach lies in its simplicity and straightforward application for one who wants to calibrate a magnetic tweezers instrument without using sophisticated custom-written routines to correct for camera-based detection biases^39,41–43^. The recent development of bright illumination sources^57^ enables the force calibration based on the equipartition theorem, as very short *τ*_*sh*_ are now accessible independently of the camera maximum frame rate or by illumination intensity fast modulation^46^. In a recent study^42^, one magnets - magnetic bead - tether configuration, i.e. 1 mm gap vertically aligned magnets and M270 magnetic beads tethered by a ~20.6 kbp dsDNA construct, has been used to perform a force calibration with *τ*_*sh*_ = 1 ms and *f*_*ac*_ = 100 Hz. The authors demonstrated a good agreement between the correction-free force calibration using Equation (1) and the calibration using the image blurring-corrected power spectral density method^39^. However, the experimental conditions at which the correction-free calibration must be performed were not provided, which become critical for more stringent experimental conditions that lead to smaller *t*_*c*,*x*_, e.g. smaller magnetic beads (Fig. 2c) or smaller nucleic acid constructs (Supplementary Fig. 3). Furthermore, it was mentioned that, to obtain the same statistical error, the correction-free method requires larger data sets, i.e. longer experiments, than the bias-corrected methods, making low forces measurement hardly accessible to the correction-free method. However, given that the statistical error only decreases by cumulating uncorrelated data points, the statistical error of the measurement does not depend on the value of *τ*_*sh*_ compared to *f*_*ac*_, i.e. either ${\tau }_{sh}\sim 1/{f}_{ac}$ (zero dead time) or ${\tau }_{sh} < 1/{f}_{ac}$ (non-zero dead time), but only on the number of cumulated uncorrelated points. In another study, the torsional stiffness of a magnetic tweezers trap was extracted using the equipartition theorem^32^, assuming that having *t*_*c*,*x*_ ~ 3-fold larger than *τ*_*sh*_ should be sufficient to obtain a variance measurement within ~10% error. We actually show here that 4-fold excess is sufficient to reach a ~10% (s.d.) error (Fig. 2c), in agreement with the theoretical prediction (Equation (3)), and we recommend the reader to not go beyond if the illumination intensity threatens the force estimation by increasing the tracking noise. We provide here an accurate evaluation of the experimental conditions to employ directly the equipartition theorem at small *τ*_*sh*_ and low *f*_*ac*_, which was lacking to the best of our knowledge. Because of their homogeneity in force and size, we highly recommend the use of MyOne and M270 magnetic beads to perform single-molecule magnetic tweezers experiments. We believe that this method is also suitable for other force spectroscopy assays, e.g. stretch flow assays^49^ or acoustic-force spectroscopy^50^, and will therefore be useful to the community.

## Materials and Methods

### Preparation of 2.1 and 20.6 kbp dsDNA constructs

We have assembled two DNA constructs of different lengths, i.e. 20.6 kbp and 2.1 kbp. The two constructs are made of a dsDNA stem flanked by two handles, which are either biotin- or digoxigenin-labeled. The dsDNA stem of the 20.6 kbp DNA construct (length: 20666 bp) is obtained by digesting the Supercos1, λ1, 2 plasmid (kindly provided by Dr. Jan Lipfert) with the restriction enzymes *Not*I and *Xho*I. The two handles are produced by PCR amplification of a 850 bp λ-DNA fragment (ThermoFisher Scientific, USA) (Primers: forward AAAAGCGGCCGCCCAGCGAGTCACTCAGCGC and reverse AAAACTCGAGTCTGCTGCTCAGCCTTC) in the presence of either digoxigenin-11-dUTPs or biotin-16-dUTPs (Jena Bioscience, Germany) to a final concentration of 40 µM. The biotin and the digoxigenin handles are digested by *Xho*I and *Not*I, respectively, and subsequently ligated to the stem with T4 DNA ligase (New England Biolabs, USA)^58^.

The dsDNA stem of the 2.1 kbp DNA construct is obtained by PCR using the pMTT2 plasmid (pMK-T derivative *with 50*% *GC random 2*.*1* *kb insert*, ThermoFisher Scientific) as a template (Primers: forward AAAATAGGAGAGACCCTTTGGATCCCGTCATTGCG and reverse AAAAGGTCTCTGCAAAGTAAAGCTTAGTGTCACGC). The two handles are produced by PCR amplification of a 435 bp λ-DNA fragments (Primers: forward AAAACCTAAGAGACCGGAACCAAAGGATATTCAGACG and reverse AAAAGGTCTCATTGCGGATCCCGTGATGACCTC) with either biotin or digoxigenin labeled dUTPs added to the PCR reaction. The stem and the handles are digested with *Bsa*I and subsequently ligated as described above.

All PCR, endonuclease restriction reactions and final ligation reactions are purified using the QIAquick PCR Purification Kit (Qiagen). The primers are obtained from Biomers.net GmbH.

### Magnetic tweezers experimental configuration

The magnetic tweezers apparatus is implemented on a custom-built inverted microscope that has already been described elsewhere^36,37^ (Fig. 1a). The collimated light emitted by an LED (660 nm, 400 mW, LH CP7P, Hechigen, Germany; spherical condenser, NA = 0.79, Thorlabs, Germany) illuminates the sample through the gap between the magnets pair^57^. The applied magnetic field is generated by a pair of vertically aligned permanent magnets (neodymium 5 mm cubes, W-05-G, SuperMagnete, Switzerland)^33^ separated by a gap of either 0.3 or 1 mm, from which the vertical distance to the sample and the rotation are controlled by two linear motors, respectively M-126.PD1 and CD-150 respectively controlled by two USB Mercury controllers (Physik Instrumente, Germany). The field of view is imaged by a 50x oil immersion objective (CFI Plan Achro 50 XH, NA 0.9, Nikon, Germany) that is mounted onto a P-726 PIFOC piezo stage controlled by the E-753 piezo controller (Physik Instrumente, Germany). The image is formed by an achromatic doublet (f = 200 mm, Thorlabs, Germany) onto a CMOS camera (Dalsa Falcon2 FA-80-12M1H, Stemmer Imaging, Germany), which is controlled by the PCIe 1433 frame grabber (National Instrument, USA). The CMOS camera has a field of view of 4096 × 3072 pixels and 6 µm pixel size. The magnetic tweezers apparatus interface and the CPU or GPU-based tridimensional bead position tracking algorithm using are written in a custom software (LabView 2016, National Instruments, USA)^36^. For GPU-based tracking, we used a GeForce GTX 1080 (NVIDIA) graphic card.

### Flow cell assembly and preparation

The flow cells are made of a double-layer parafilm (Parafilm^®^M, P7793, Sigma Aldrich, Germany) sandwiched by two microscope glass coverslips (#1, 24 × 60 mm, Menzel GmbH, Germany). The top coverslip have two holes drilled using a sandblaster (Problast 2, Vaniman, CA, USA) with Al_2_O_3_ particles (34–82 µm, F230, Eisenwerk Wuerth, Germany). The coverslips are washed by sonication in a 2% (V/V) Hellmanex III (Sigma Aldrich, Germany) aqueous solution for 15 minutes at 40 °C, and subsequently thoroughly rinsed with deionized water and dried at ~80 °C. ~4 µl of nitrocellulose solution (0.1% (m/V) in amylacetate (Sigma Aldrich, Germany)) is spread on the top side of the bottom coverslips (the inner bottom side of the flow cell). The double-layer Parafilm is carved with a scalpel to form a channel, sandwiched between the two coverslips such that the two holes of the top coverslips are aligned within the channel, and melted at ~90 °C for ~30 seconds. For the thin flow cells making, the #1 top coverslip is replaced by a #0 coverslip of the same dimension and the channel is carved into a stretched single-layer of Parafilm, reducing the flow cell height by ~200 µm.

After mounting the flow cell on the microscope, ~1 ml of rinsing buffer — 10 mM HEPES, 100 mM NaCl, pH 7.4 (Roth, Germany) — is flushed in the flow cell using a peristaltic pump (Reglo, Ismatec, Germany), and either 1.1 or 3 µm polystyrene beads (LB11, LB30 respectively, Sigma Aldrich, Germany) (diluted 1:1500 in measurement buffer) are non-specifically attached to the surface during a 10 min incubation to serve as reference beads to correct for the flow chamber mechanical drift. The surface is then functionalized by incubating for 30 minutes anti-digoxigenin (Roche, Switzerland) diluted to a final concentration of 50 µg/ml in rinsing buffer. The flow chamber is finally passivated by incubating bovine serum albumin (BSA) (New England Biolabs, USA) diluted to a final concentration of 10 mg/ml in rinsing buffer for 30 minutes. Between steps, the flow cell is rinsed with ~1 ml of rinsing buffer. 100 µg of MyOne or 200 µg of M270 streptavidin-coated superparamagnetic Dynabeads (Thermofisher, Germany) are washed twice in measurement buffer (rinsing buffer supplemented with 0.1 mg/ml BSA), and finally incubated with ~15 pM of DNA in measurement buffer. The DNA-bound beads are then incubated for ~15 minutes in the flow chamber to attach the magnetic beads to the top surface of the bottom coverslip. Finally, the superfluous magnetic beads are removed by flushing through ~2 ml of measurement buffer. Nonspecifically and weekly attached magnetic beads are then removed from the flow chamber bottom surface by applying forces up to ~30 pN for M270 magnetic beads and ~8 pN for MyOne magnetic beads, while rotating the magnets back and forth and gently tapping the outlet capillary tubes (1 mm id, PEEK, Upchurch Scientific, USA), and repeating this procedure over the microfluidic channel from the inlet to the outlet. The non-tethered beads are then flushed out with ~1–2 ml of measurement buffer.

### Bead selection, measurement and analysis

To select for singly tethered magnetic beads, we measure changes in extension for the 20.6 kbp DNA construct while rotating the magnets from −100 to +100 turns at ~3 pN constant force. With coilable molecules, *L*_*ext*_ remains unchanged when the DNA is negatively supercoiled, and *L*_*ext*_ decreases with the increase of positive supercoils (once the DNA molecule has passed the buckling transition). For non-coilable molecules, *L*_*ext*_ is unchanged independently of the sign and the number of magnets turns. Finally, muti-tethered magnetic beads position decreases for positive and negative magnet turns^6^. For the high force measurement with M270 magnetic beads, the experiments are performed with only coilable molecules, to avoid the overstretching transition above ~50 pN^59^ (both strand of the handles are labeled in the DNA constructs used here). To avoid the side tethering effect at low force^43,55^, we have selected the tethers such as their lengths had to be within ~10% of the contour length at the 8 pN force and zero magnets turn.

*f*_*ac*_ is 10 Hz, unless otherwise specified. *τ*_*sh*_ is set as indicated and the illumination intensity is adjusted prior to each measurement to ~160 grey levels (256 total grey levels). For each magnets position, we acquire 500 points separated by a time interval larger than *t*_*c*,*x*_ for the estimated applied force and tether length. For *t*_*c*,*x*_ larger than the time between subsequent openings of the camera shutter, e.g. *t*_*c*,*x*_ > 100 ms, the duration of the acquisition is extended to a total period of $500\cdot {t}_{c,x}$ and the data points acquired in the interval defined by the correlation time are filtered out during the analysis.

### Theoretical consideration to calibrate the force in a magnetic tweezers experiment

In a magnetic tweezers experiment, the DNA tether experiences a magnetic force *F* along the z-axis, which is counteracted by the restoring force of the tethered DNA molecule. The bead fluctuates in position along the three axes, experiencing the Brownian motion due to the impact of the water molecules in the solution. Neglecting the inertia contribution, the equations of motion of the magnetic bead along the x- and z-axis are:

$$6\pi \eta R\cdot \dot{x}(t)+{k}_{x}\cdot x(t)+{F}_{x}^{WLC}={F}_{therm}+F$$

$$6\pi \eta R\cdot \dot{z}(t)+{k}_{z}\cdot z(t)+{F}_{z}^{WLC}={F}_{therm}+F-{F}_{grav}$$

where *k* is the stiffness of the trap, *η* is the viscosity of the medium, *R* is the radius of the tethered magnetic bead, *F*_*therm*_ is the thermal (or Langevin) force, *F*^*WLC*^ is the restoring force on the DNA tether, and *F*_*grav*_ is the gravitational force on the bead^37,54^. To take into account the effect of the proximity between the flow chamber surface and the tethered bead, *R* is corrected using the Faxén law^37^. To generate the numerical simulations to evaluate our experiments, we solve the above equations with a finite-difference time-stepping algorithm, which has been previously described^37,54^. We calculate *t*_*c*,*x*_ and evaluate against the corner frequency $\,{f}_{c,x}=1/(2\pi {t}_{c,x})$ obtained from the power spectral density fitted by the LabVIEW software routine provided by Daldrop and coworkers^43^. For M270 magnetic beads, the 20.6 kbp DNA construct, a 30 pN applied force, and acquiring the data at *f*_*ac*_ = 1720 Hz with ${\tau }_{sh}=1/{f}_{ac}$, we measure from the data *t*_*c*,*x*_ = 5.86 ms and we calculate *t*_*c*,*x*_ = 5.62 ms. Similarly, for MyOne magnetic beads, the 2.1 kbp DNA construct, a 6 pN applied force, and acquiring the data at *f*_*ac*_ = 1720 Hz with ${\tau }_{sh}=1/{f}_{ac}$, we measure from the data *t*_*c*,*x*_ = 1.28 ms and we calculate *t*_*c*,*x*_ = 0.95 ms. Overall, Equation (2) gives a good estimate of the characteristic timescale of the system, and we therefore apply it to evaluate *t*_*c*,*x*_ throughout the study.

### Evaluating the relative error on 〈*δx*^2^〉 from using a detector with a finite bandwidth *f*_*ac*_/2

The theoretical expression of the relative error in the variance of the tethered bead position when using a detector with a finite bandwidth *f*_*ac*_/2 is^4,53^:

$$\langle \delta {x}_{eq}^{2}\rangle ={\int }_{0}^{{f}_{ac}/2}\,{S}_{x}(f)df=\frac{2{k}_{B}T}{\pi {k}_{x}}\,\arctan \,(\frac{{f}_{ac}}{2\cdot {f}_{c,x}})$$

where ${S}_{x}(f)={k}_{B}T/\gamma {\pi }^{2}({f}_{c,x}^{2}+{f}^{2})$ is the power spectral density of the bead position along the x-axis.

For $${f}_{ac}\sim \infty $$,

$$\langle \delta {x}^{2}\rangle =\frac{{k}_{B}T}{{k}_{x}}$$

Therefore,

$$\langle \delta {x}_{eq}^{2}\rangle /\langle \delta {x}^{2}\rangle =\frac{2}{\pi }\,\arctan \,(\frac{{f}_{ac}}{2\cdot {f}_{c,x}})$$

The above expression is modified such as:

3 $$\frac{{F}_{meas}}{F}=\frac{\langle \delta {x}^{2}\rangle }{\langle \delta {x}_{eq}^{2}\rangle }=\frac{2}{\pi \cdot \arctan (\frac{\pi \cdot {t}_{c,x}}{{\tau }_{sh}})},$$

where *F*_*meas*_ is the measured force and *F* is the expected force, and is plotted in Fig. 2c.

## Electronic supplementary material

Supplementary Information

## Data Availability

The data of this study are available upon reasonable request from the authors.
