# Author Correction: Correction-free force calibration for magnetic tweezers experiments

**DOI:** 10.1038/s41598-018-36219-0

**Published:** 2018-12-10

**Authors:** Eugen Ostrofet, Flávia Stal Papini, David Dulin

**Affiliations:** Junior Research Group 2, Interdisciplinary Center for Clinical Research, Friedrich Alexander University Erlangen-Nürnberg (FAU), Hartmannstr. 14, 91052 Erlangen, Germany

Correction to: *Scientific Reports* 10.1038/s41598-018-34360-4, published online 29 October 2018

The original version of this Article contained errors in the Abstract.

“By significantly reducing τsh to at least 4-fold the characteristic times of the tethered magnetic bead, we accurately evaluated the variance of the magnetic bead position along the axis parallel to the magnetic field, estimating the force with a relative error of ~10% (standard deviation), being only limited by the bead-to-bead difference.”

now reads:

“By significantly reducing τsh to at most 1/4 the characteristic time of the tethered magnetic bead, we accurately evaluated the variance of the magnetic bead position along the axis parallel to the magnetic field, estimating the force with a relative error of ~10% (standard deviation), being only limited by the bead-to-bead difference.”

These errors have now been corrected in the PDF and HTML versions of the Article.

