# Supplementary material for: Correction-free force calibration for magnetic tweezers experiments
Source: Sci Rep. 2018 Oct 29;8:15920. doi: 10.1038/s41598-018-34360-4 (PMC6206022; doi:10.1038/s41598-018-34360-4)
Supplement: Supplementary file 1 — Supplementary Information [file 41598_2018_34360_MOESM1_ESM.pdf]

Supplementary Information for:

Correction-free force calibration for magnetic tweezers experiments

Eugen Ostrofet, Flávia Stal Papini, David Dulin*

Junior Research Group 2, Interdisciplinary Center for Clinical Research, Friedrich Alexander University Erlangen-Nürnberg (FAU), Hartmannstr. 14, 91052 Erlangen, Germany.

*Correspondence may be addressed to: david.dulin@uk-erlangen.de

Supplementary Information contains Supplementary Figure S1-S4.

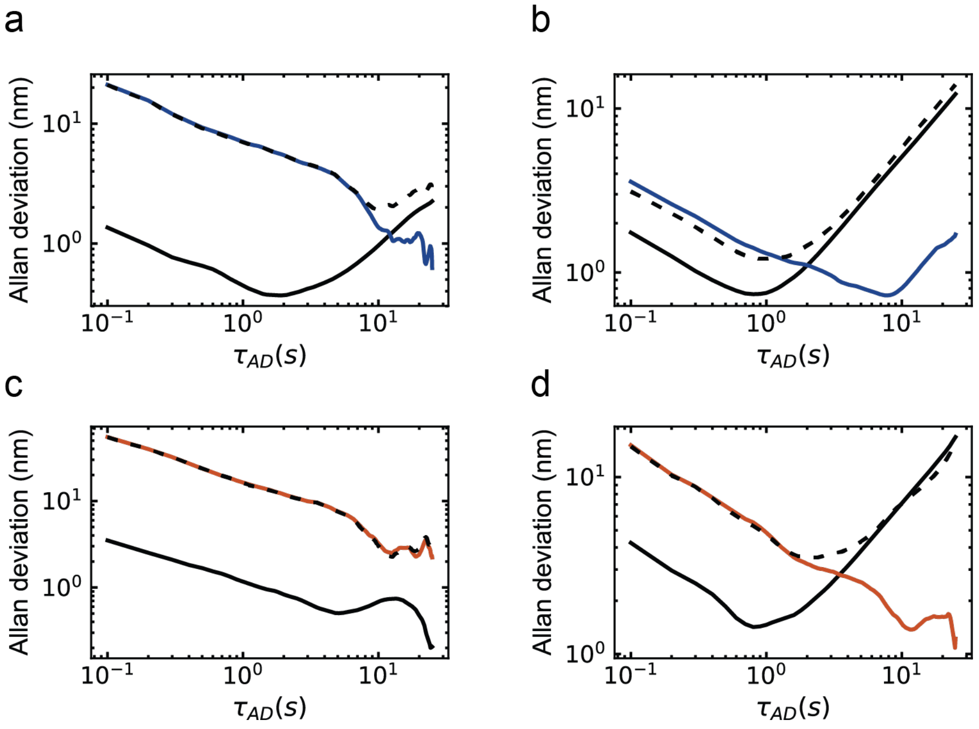

Supplementary Figure 1: Spatiotemporal resolution of the magnetic tweezers apparatus. (a) Allan deviations for the M270 magnetic bead tethered by the 20.6 kbp DNA construct at ~ 60 pN applied force with (solid blue line) or without (dashed black line) drift correction using a 3 µm reference bead (solid black line) along the x-axis and (**b**) the z-axis. **(c)** Allan deviations for the MyOne magnetic bead tethered by the 20.6 kbp DNA construct at ~ 8 pN applied force with (solid orange line) or without (dashed black line) drift correction using a 1.1 µm reference bead (solid black line) along the x-axis and (**d**) the z-axis.

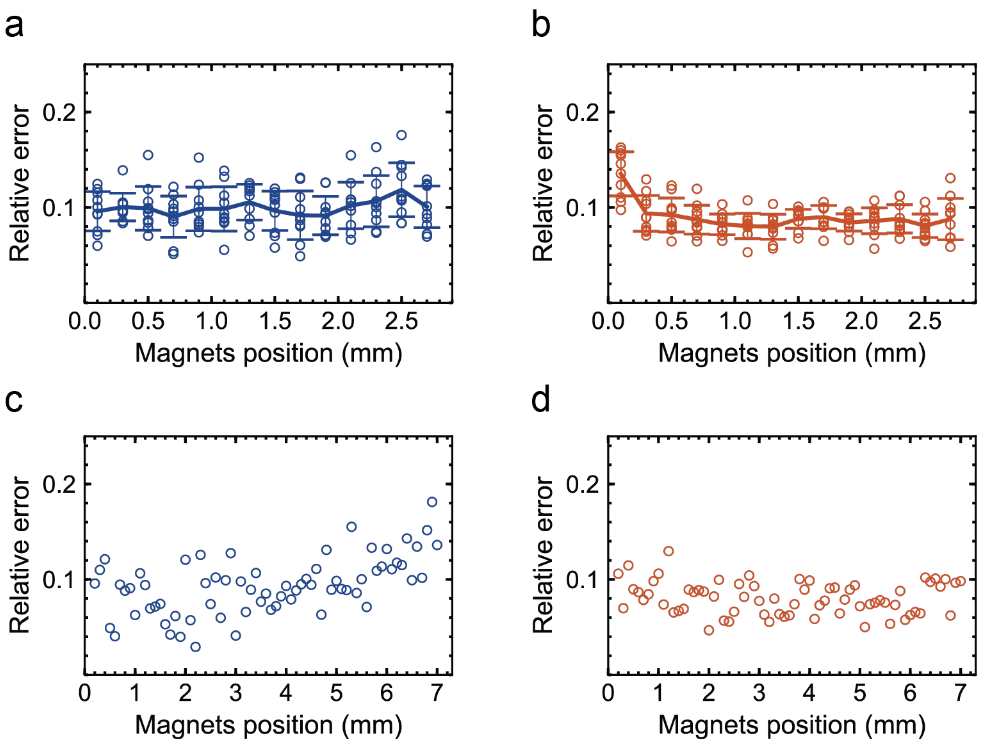

Supplementary Figure 2: Force estimation accuracy. The relative error is the standard deviation of the force divided by the average force measured for several beads. At each magnets position (0 mm is the top surface of the top coverslip of the flow chamber), each circles represent the relative error for a force estimation at a given τ_{sh} (**Figure 2ab**), using either (**a**) M270 or (**b**) MyOne magnetic beads. The error bars are one standard deviation of the relative errors distribution, and the solid lines represent the average relative errors. (**c**) The circles represent the relative error for each magnets position in the force estimation of **Figure 3a** for M270 magnetic beads. (**d**) Same plot as in (c) for MyOne magnetic beads.

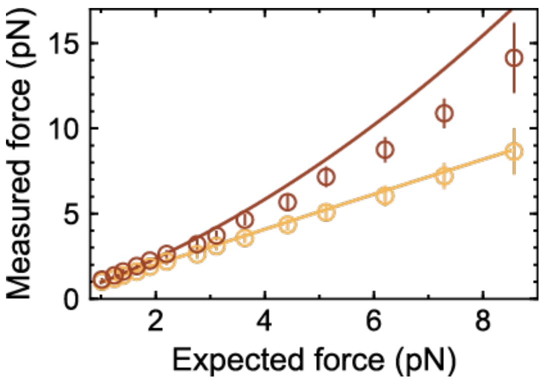

Supplementary Figure 3: Comparison of numerical simulation and experiments for force calibration with MyOne magnetic beads and two camera open shutter times. Measured force from experiments (circles) and numerical simulations (solid lines, **Material and Methods**) as a function of the expected force, for the 20.6 kbp DNA construct, MyOne magnetic beads (1 µm diameter), 1 mm gap vertically oriented magnets configuration, and $\tau_{sh} = 1$ ms (yellow) and $\tau_{sh} = 20$ ms (brown). The error bars are one standard deviation.

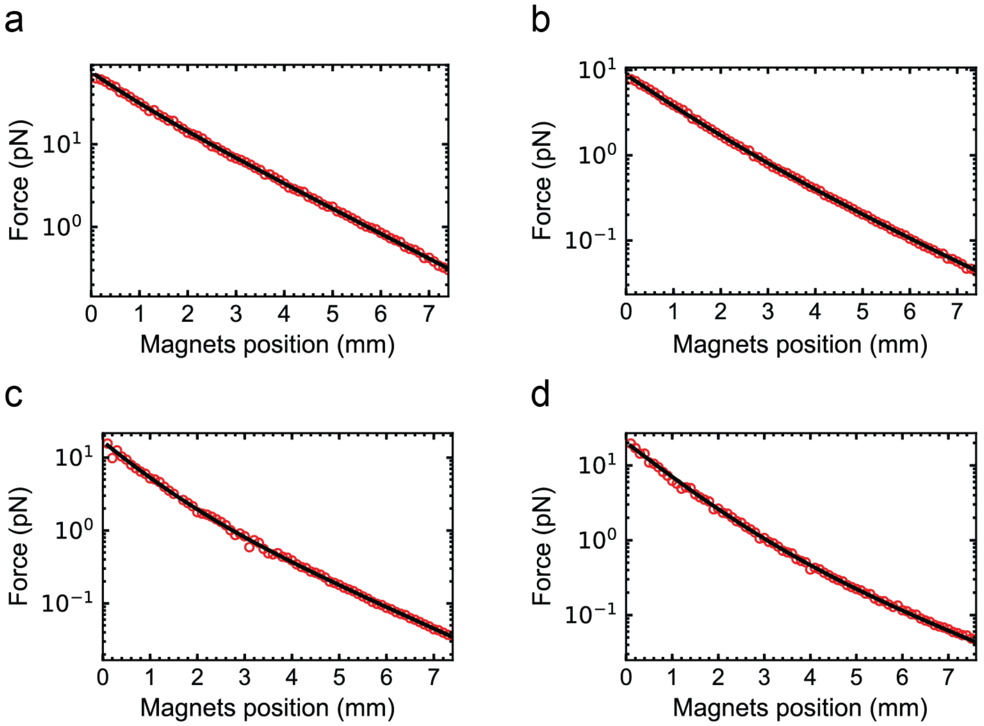

Supplementary Figure 4: Force estimations and bi-exponential phenomenological fits. Forces estimated from the equipartition theorem with an image acquisition at $\tau_{sh} = 0.5$ ms (red circles) plotted against the magnets position for **(a)** M270 magnetic beads, for a 1 mm gap vertically aligned magnets configuration; n = 6. **(b)** MyOne magnetic beads, for a 1 mm gap vertically aligned magnets configuration; n = 11. **(c)** MyOne magnetic beads, for a 0.3 mm gap vertically aligned magnets configuration; n = 8. **(d)** MyOne magnetic beads, for a 0.3 mm gap vertically aligned magnets configuration, thin flow cell; n = 6. The solid lines are extracted from a bi-exponential fit to the data (mathematical expressions detailed in **Table 1**).
